# Supplementary material for: Neural and computational processes underlying dynamic changes in self-esteem
Source: eLife. 2017 Oct 24;6:e28098. doi: 10.7554/eLife.28098 (PMC5655144; doi:10.7554/eLife.28098)
Supplement: Supplementary file 1 [file elife-28098-supp1.docx]

***Supplementary file 1.*** *Instructions and questions for online profile on which participants were evaluated.*

You will now create a profile about yourself. You will create your profile via this online platform, so that we can show it to other people taking part in the study. These people are men and women between the ages of 18 and 30. They will be asked what they think about you. They can choose to like or dislike you.

This is how they are asked make their decision:

*“Do you like this person? Do you think you could you be friends with them in real life? Or do you think this person is boring and are you not interested in getting to know them any better?*

*Press the “like” button, if you think you could be friends with this person in real life. Press the “dislike” button if you are not interested in getting to know them.”*

Your profile will be made up of your answers to 5 personal questions. Together they will give others a good idea about who you are. Please answer each question thoughtfully and honestly in roughly 2-3 sentences. Take the time to answer, because this is the only information that others will have to judge you.

Questions:

1. If we asked your friends and family about your best qualities, what might they say?

2. And what would they say were your worst qualities?

3. What are you most afraid of?

4. My favorite things in life are:

5. I really dislike people who (for example people who are lazy, mean to others, or make annoying noises when they eat):
