# Supplementary material for: Neural and computational processes underlying dynamic changes in self-esteem
Source: eLife. 2017 Oct 24;6:e28098. doi: 10.7554/eLife.28098 (PMC5655144; doi:10.7554/eLife.28098)
Supplement: Supplementary file 2. [file elife-28098-supp2.docx]

Supplementary table 1
*Brain regions revealed by whole-brain regression analysis with trial-by-trial social approval prediction errors as parametric modulator at the onset of feedback presentation (thresholded at* p *< .001 uncorrected, k > 50 voxels).*

| Brain region | L/R | k | t | MNI coordinates | | |
| --- | --- | --- | --- | --- | --- | --- |
|  |  |  |  | x | y | z |
| Ventral Striatum/sgACC | R | 493 | 5.42 | 5 | 20 | -8 |
|  | L |  | 4.50 | -8 | 21 | -5 |
|  | L |  | 4.17 | -8 | 8 | -15 |
| Parahippocampal gyrus | L | 200 | 5.27 | -32 | -24 | -18 |
| Precentral gyrus | L | 278 | 4.84 | -42 | -9 | 63 |
| Superior frontal gyrus | L | 97 | 4.58 | -20 | 32 | 41 |
| Inferior frontal gyrus | L | 51 | 4.09 | -44 | 33 | 9 |
| Medial orbitofrontal cortex | L | 114 | 3.85 | -3 | 62 | -2 |

*Note.* L/R=Peak in Left/Right hemisphere; k=cluster size in 1.5×1.5×1.5mm voxels; Z=z-score; MNI coordinates =xyz voxel coordinates in MNI space of the peak voxel. ACC = anterior cingulate cortex

Supplementary table 2

*Brain regions revealed by whole-brain regression analysis with trial-by-trial self-esteem updates (inferred using our computational model) as parametric modulator at the onset of feedback presentation (thresholded at* p *< .001 uncorrected, k > 50 voxels).*

| Brain region | L/R | k | t | MNI coordinates | | |
| --- | --- | --- | --- | --- | --- | --- |
|  |  |  |  | x | y | z |
| Superior temporal gyrus | L | 82 | 3.77 | -50 | -39 | 17 |
| Ventromedial prefrontal cortex | L | 81 | 3.73 | -5 | 48 | -2 |
|  | L |  | 3.55 | -2 | 42 | -9 |

*Note.* L/R=Peak in Left/Right hemisphere; k=cluster size in 1.5×1.5×1.5mm voxels; Z=z-score; MNI coordinates =xyz voxel coordinates in MNI space of the peak voxel.

Supplementary table 3
*Brain regions revealed by whole-brain regression analysis testing for inter-individual differences in “interpersonal vulnerability” (between-subjects regressor) in responses to social approval prediction errors (thresholded at* p *< .001 uncorrected, k > 50 voxels).*

| Brain region | L/R | k | t | MNI coordinates | | |
| --- | --- | --- | --- | --- | --- | --- |
|  |  |  |  | x | y | z |
| Cerebellum (VI) | L | 720 | 5.90 | -6 | -71 | -20 |
| Fusiform gyrus | L | 206 | 5.73 | -32 | -41 | -20 |
| Middle temporal gyrus | L | 2474 | 5.33 | -39 | -50 | 11 |
| Superior occipital gyrus | R | 1216 | 5.26 | 26 | -98 | 12 |
| Calcarine gyrus | L | 1002 | 4.99 | -5 | -60 | 9 |
| Superior frontal gyrus | R | 64 | 4.99 | 18 | 17 | 50 |
| Inferior temporal gyrus | R | 100 | 4.87 | 39 | 3 | -42 |
| Middle temporal gyrus | R | 722 | 4.84 | 51 | -74 | 14 |
| Rolandic operculum | R | 255 | 4.74 | 53 | 2 | 17 |
| Anterior cingulate cortex | L | 300 | 4.74 | -8 | 36 | 29 |
| Anterior insula/inferior frontal gyrus | L | 339 | 4.70 | -44 | 11 | 9 |
| Medial frontal gyrus | L | 149 | 4.68 | -5 | 65 | 6 |
| Medial orbitofrontal cortex | L | 330 | 4.67 | -18 | 27 | -15 |
| Inferior frontal gyrus/orbitofrontal cortex | R | 640 | 4.47 | 26 | 12 | -20 |
| Cerebellum (IX) | L | 153 | 4.42 | -9 | -47 | -33 |
| Inferior temporal gyrus | R | 82 | 4.36 | 54 | -21 | -24 |
| Precuneus | L | 104 | 4.33 | -9 | -51 | 45 |
| Superior occipital gyrus | L | 80 | 4.29 | -8 | -98 | 21 |
| Superior frontal gyrus | L | 169 | 4.20 | -12 | 57 | 35 |
| Cerebellum (VIII) | R | 133 | 4.18 | 12 | -72 | -45 |
| Posterior cingulate cortex | R | 50 | 4.17 | 5 | -39 | 14 |
| Middle occipital gyrus | L | 195 | 4.15 | -29 | -86 | 6 |
| Inferior frontal gyrus | R | 101 | 4.15 | 57 | 21 | 14 |
| Middle temporal gyrus | L | 68 | 4.13 | -57 | 2 | -27 |
| Superior temporal gyrus | L | 119 | 3.89 | -48 | -18 | -6 |
| Precuneus | R | 243 | 3.88 | 3 | -44 | 44 |
| Putamen | L | 82 | 3.86 | -12 | 11 | -5 |
| Cerebellum (VII) | L | 74 | 3.77 | -12 | -74 | -44 |
| Superior frontal gyrus | L | 58 | 3.72 | -23 | 48 | 39 |

*Note.* L/R=Peak in Left/Right hemisphere; k=cluster size in 1.5×1.5×1.5mm voxels; Z=z-score; MNI coordinates =xyz voxel coordinates in MNI space of the peak voxel. ACC = anterior cingulate cortex

Supplementary table 4
*Brain regions revealed by whole-brain regression analysis testing for inter-individual differences in “interpersonal vulnerability” (between-subjects regressor) in functional coupling between the anterior*

*insula (-44, 11,9) and the rest of the brain during self-esteem updates (thresholded at* p *< .001 uncorrected, k > 50 voxels).*

| Brain region | L/R | k | t | MNI coordinates | | |
| --- | --- | --- | --- | --- | --- | --- |
|  |  |  |  | x | y | z |
| Medial prefrontal cortex (including dorsal ACC, | L | 16947 | 6.53 | -12 | 26 | 36 |
| medial orbitofrontal cortex (BA 14m), and | R |  | 6.27 | 11 | 32 | -11 |
| medial orbitofrontal cortex (BA 11m) | R |  | 5.35 | 3 | 62 | -12 |
| Medial temporal pole | R | 10014 | 6.18 | 41 | 9 | -36 |
| Posterior cingulate cortex | L | 1836 | 6.08 | -2 | -45 | 15 |
| Inferior frontal gyrus | L | 193 | 5.51 | -36 | 41 | -6 |
| Inferior frontal gyrus | R | 431 | 5.22 | 56 | 38 | -11 |
| Middle temporal gyrus | L | 2957 | 4.99 | -60 | -11 | -12 |
| Orbitofrontal cortex | L | 196 | 4.84 | -20 | 26 | -27 |
| Middle frontal gyrus | R | 485 | 4.82 | 35 | 24 | 38 |
| Inferior frontal gyrus | R | 264 | 4.79 | 50 | 24 | 14 |
| Fusiform gyrus | L | 529 | 4.75 | -24 | -30 | -23 |
| Precentral gyrus | R | 3038 | 4.59 | 39 | -12 | 59 |
| Cerebellum (Crus 1) | R | 107 | 4.44 | 41 | -68 | -33 |
| Angular gyrus | L | 613 | 4.38 | -44 | -72 | 36 |
| Cerebellum (VI) | L | 362 | 4.21 | -9 | -62 | -14 |
| Superior temporal gyrus | R | 147 | 4.20 | 44 | -33 | 3 |
| Middle frontal gyrus | L | 74 | 4.15 | -35 | 23 | 38 |
| Superior Parietal lobule | R | 94 | 4.01 | 33 | -65 | 59 |
| Fusiform gyrus | L | 121 | 3.98 | -44 | -68 | -17 |
| Precentral gyrus | R | 92 | 3.95 | 53 | 0 | 26 |
| Rolandic operculum | R | 105 | 3.85 | 36 | -21 | 17 |
| Postcentral gyrus | R | 87 | 3.83 | 35 | -36 | 63 |
| Angular gyrus | L | 51 | 3.48 | -48 | -54 | 29 |

*Note.* L/R=Peak in Left/Right hemisphere; k=cluster size in 1.5×1.5×1.5mm voxels; Z=z-score; MNI coordinates =xyz voxel coordinates in MNI space of the peak voxel. ACC = anterior cingulate cortex
